# The possible adaption of the human respiratory system to past atmospheres

**DOI:** 10.1113/EP091713

**Published:** 2024-03-27

**Authors:** Alan Cannell

**Affiliations:** ^1^ Institute of Advanced Studies (Human Evolution) University of São Paulo São Paulo Brazil; ^2^ ISIPU Istituto Italiano di Paleontologia Umana Rome Italy

Humans possess physiological systems that have evolved over tens or even hundreds of millions of years and adapted to the changing conditions of Earth. As a result, we share many ancient characteristics with other life forms; for example, above ∼42°C the proteins in the human body break down and such temperatures are fatal—as they are for dogs, cattle, giant hornets (*Vespa velutina nigrithorax*) (Ruiz‐Cristi et al., 2020) and large dragonflies (May, 1982). The crow‐sized griffenflies and other giant volant insects of the Permian probably had similar thermoregulatory limits and could only function as predators due to a much denser, oxygen‐rich air which allowed flight at lower power and heat outputs; ground beetles, on the other hand, had to overcome a constant gravity with six legs and were thus their present size (Cannell, 2018; Cannell & Nel, 2023). Blood pressure for over 40 mammalian species also shows that there is no strong correlation with body mass, even across four orders of magnitude, presumably reflecting a basal value that has been adopted for many millions of years, the exception being the giraffe, which has a heart output pressure about twice that of humans (Miller, 2018).

Most land mammals have similar respiratory systems that can support high altitude hypoxia of ∼0.6 bar (450 mmHg), allowing a greater range of altitudes to be populated. What is surprising, however, is that humans (and dogs) can tolerate hyperbaric pressures of ∼1.9 bar (i.e., breathing compressed air at a water depth of 9 m) with no harmful effects in terms of nitrogen absorption or oxygen toxicity. It is possible to dive much deeper with aponia, but we cannot breathe beyond a depth of 9 m without the need for decompression. Saturation deep sea divers tolerate long periods of breathing Heliox (helium–oxygen), but again, only provided that the partial pressure of oxygen is kept below 0.4 bar. The human respiratory system thus appears to have been ‘engineered’ to function between pressures of 0.6 and 1.9 bar and a partial pressure of oxygen between 0.12and 0.40 bar (where the present value is 0.21 bar) The lower tolerance values can be explained in terms of the need to traverse or populate high altitude zones, but why are we able to tolerate higher pressures? One possible reason is that air pressure and density might not be constant over time and very long‐term changes over tens of millions of years are mirrored in these tolerances.

Many attempts have been made to model the evolution of oxygen in Earth's atmosphere over the past 400 Myr. There have variously been used isotope mass balance models (Royer et al., 2014); the evidence of wildfires in the geological record (Glasspool et al., 2015); the relation of carbon isotopes in plant resins (Tappert et al., 2013); the redox characteristics of framboidal pyrites (Cannell et al., 2022; Large et al., 2019); direct measurements of oxygen in halite (Brand et al., 2021); and the respiration constraints of large dragonfly insects bauplan (Cannell & Nel, 2023).

All mass‐derived box models for estimating past oxygen, and by extension all climate models, assume a constant mass of atmospheric nitrogen for examining deep time, although there is no evidence for this assumption. The main reservoir of N_2_ is now considered to be in the lithosphere and mantle (Johnson & Goldblatt, 2015, 2018), and the assumption that atmospheric nitrogen is constant over hundreds of millions of years is no longer tenable. Some estimates of past oxygen levels resulted in very low values for the Mesozoic and Cenozoic and were thus initially rejected by the scientific community as being incompatible with the accepted minimum value of oxygen for wildfires to propagate. Again, this is only true in the present atmosphere, as wildfires reflect the relation between air pressure and oxygen levels (Harper et al., 2016; McAllister et al., 2010; West, 2001), invalidating the constraints implied in charcoal‐based models such as Glasspool et al. (2015). On the other hand, macro charcoal wildfire data (or lack of) do serve as an important proxy for paleoatmospheres. For example, when there is a ‘charcoal gap’ in the record, such as the Devonian from about 375 to 363 Mya, or the early Triassic (Jasper et al., 2021), and reliable proxies for the partial pressure of oxygen are available, the lack of macro charcoal from wildfires allows a limit for paleo sea‐level air pressure to be derived.

The evolution of flight and the size of flying animals is also a useful proxy for paleo air density. Muscle power, takeoff airspeeds, thermoregulation and respiration can be modelled suggesting that the giant volant insects of the Permian required ∼2 bar to function (Cannell & Nel, 2023); the Miocene giant birds probably flew in air at 1.3 bar (Cannell, 2020) and bats evolved at 1.6 bar (Giannini et al., 2024). Again, the ‘hexapod gap’, around 380–320 Mya, a period in which there is a paucity of winged insect fossils (Schachat et al., 2018), suggests a low‐density atmosphere in which gliding and subsequent flapping flight could not efficiently develop (Cannell et al., 2022).

In engineering terms, a tree can be viewed as a column of water, and cohesion–tension theory suggests that at higher air pressures this column can be taller. Canopy heights have been derived as being 20–30 m at 430, 390, 350 and 310 Mya, with lows that match other atmospheric limitations at 380−370, 355 and 335 Mya (Retallack, 2022). The ancient *Araucaria* conifers of southern South America today reach heights of between 20 and 30 m, but in the late Triassic thousands of fossilized trunks of *Araucarioxylon arizonicum* were preserved in Arizona with living heights of several trunks estimated to be up to 59 m. The most spectacular fossil *Araucaria mirabilis* trunks are seen at the Cerro Cuadrado Petrified Forest, Patagonia, Argentina, dated at about 160 Mya, with fallen trunks of up to 68.8 m in length but apparently possessing a very similar biology to extant species (Cúneo, 2021). How did they get this tall?

Periods of high atmospheric CO_2_, such as the Devonian, but with non‐acidic oceans (as derived from boron isotopes in brachiopods) are of particular interest. Oceans absorb a large amount of CO_2_ (currently ∼50 times that of the atmosphere) due to the relatively high solubility of this gas in seawater as expressed by Henry's constant. A reduction in atmospheric mass therefore leads to massive outgassing of fractionated CO_2_ into the air (the ‘sparkling water effect’) and relatively alkaline seas. This is seen in the Devonian, at the end Permian and the Triassic–Jurassic transition, as well as in many major cooling events that take place together with an *increase* in $P_{CO_{2}}$, as first noted by Steinthorsdottir and Vajda (2013). Conversely, an increase in atmospheric pressure increases absorption and lead to lower levels of atmospheric CO_2_, but more acidic seas, such as in the late Carboniferous and most of the Cretaceous (topic in review).

Much of the scientific community has taken for granted a constant and the extant value of atmospheric mass and the acceptance of a variable paleoatmospheric pressure is therefore a slow and on‐going process, similar perhaps to plate tectonic theory of only a few decades ago. This leads to the first question that geoscientists always ask: where does new atmosphere come from and how is it lost? Essentially O_2_ and N_2_ are a product of plate tectonics—in particular from mid‐ocean ridges and over periods of millions of years (Johnson & Goldblatt, 2018, and on‐going work). Atmospheric mass (in particular the lighter isotope ^14^N) is lost during high energy solar events in low magnetic fields and over periods of several hundred thousand years (Tsareva et al., 2022).

The quantification of paleoatmospheric density (in units of pressure, patm or bar) is still in its infancy, but a ballpark estimate can be made by considering the above proxies, all of which point to similar periods of higher and lower patm shown in Figure 1.

**FIGURE 1:**
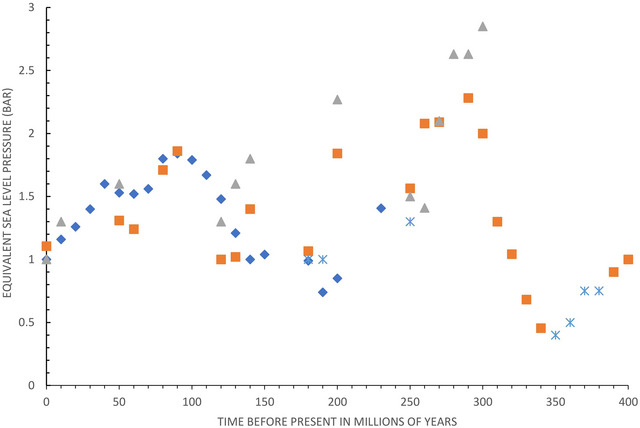
Possible variation of atmospheric pressure (bar) over time. Sources: flight, grey triangles—Cannell and Nel (2023), Cannell (2020) and Giannini et al. (2024, 2024); pyrite, proxy for $P_{O_{2}}$ (revised), orange squares—Cannell et al. (2022) and Large et al. (2019); amber, proxy for $P_{O_{2}}$ in relation to the Geocarbsulf model, blue diamonds—Royer et al. (2014) and Tappert et al. (2013); wildfire macro charcoal gap constraints, crosses—Jasper et al. (2021).

At lower density air, Hadley cells are stronger and wider (Chemke & Kaspi, 2017) and mid latitudes are drier; at higher densities, high latitude forests flourish (with magnolias and alligators thriving at the North Pole during the Eocene). Flora and fauna from the past thus provide a check on the estimates of temperature, precipitation, CO_2_ levels and paleo patm. For example, at 1 bar it has not proved possible to model either the Eocene or Miocene high latitude warm climates (Hutchinson et al., 2021; Steinthorsdottir et al., 2021).

Likewise, Neogene paleo‐elevation estimates of the Tibetan and Andean plateaux have resulted in a decades long dispute between geologists (who find that these zones were already high) and biologists (who claim that the biology was similar to species that now live at much lower altitudes: ‘proof’ that more recent massive uplift took place) (Quade et al., 2020). Both could be right, of course, except that the bio units are in bar and not in metres, low altitude patm for the late Miocene being ∼1.3 bar, as derived from amber isotopes, giant bird flight and marine boron isotopes.

In a nutshell, we now live in a cool period of lower air density, but as mammals, we evolved during periods when density was very likely higher. This possibly allows us to breathe at 1.9 bar with no serious physiological effects. Atmospheric composition and density have greatly affected the physiology of past creatures, in particular the evolution of flight and the limits of growth. Hydraulic pressures and heart pump work in very long necked titanosaurs? Extinction of all giant volant birds in the late Pliocene? Loss of all giant sharks at the same time? Appearance of bipedal hominins? These are all issues that require a consideration of past atmospheres: the one thing we can be certain of is that extinct animals did not live in extant air, although paleoatmospheres may have moulded extant life.

## AUTHOR CONTRIBUTIONS

Sole author.

## CONFLICT OF INTEREST

The author declares no conflicts of interest.

## FUNDING INFORMATION

No funding was received for this work.
